# Onabotulinum Toxin A (Botox®) in the Treatment of Neurogenic Bladder Overactivity

**DOI:** 10.5812/numonthly.1864

**Published:** 2012-03-01

**Authors:** Malene Rohrsted, Cecilie Bagi Nordsten, Per Bagi

**Affiliations:** 1 Department of Urology, Rigshospitalet, State University hospital, Copenhagen, Denmark

**Keywords:** Onabotulinumtoxin A, Urinary Bladder, overactive, Urinary Bladder, Neurogenic

## Abstract

Botulinum toxin (BT) is a potent presynaptic neuromuscular blocking agent which induces selective, reversible muscle weakness for months when injected intramuscularly. During recent years BT has revolutionized the treatment of previously intractable symptoms of detrusor overactivity. Based on a systematic search of the PubMed database, a review of the current literature on the use of onabotulinum toxin A (Botox®) in the treatment of neurogenic detrusor overactivity is presented.

Onabotulinum toxin A proved to be highly effective in the majority of studies, even though a wide range of injection techniques and dosages were described. The onset of the effect usually appeared before 2 weeks, and reached a peak within 2-6 weeks, with the clinical effect being maintained for approximately 6-8 months, or even longer. Depending on the dose, a number of patients developed high residual volume and clean intermittent self/helper catheterization (CIC) may become necessary. Only a few side effects were described, and intravesical onabotulinum toxin A injection seems to be well tolerated. However, details on injection technique, dose interval between injections, etc. are still under debate and only a few randomized, placebo controlled studies have been published.

## 1. Introduction

Urinary incontinence caused by detrusor overactivity (Do) (1) remains a major problem for many people with neurological disorders. According to the Standardization published by the International Continence Society, Do is a urodynamic observation characterized by involuntary detrusor contractions during the filling phase which may be spontaneous or provoked (1). Do may also whenever possible, be classified as neurogenic detrusor overactivity (NDo) when there is a relevant neurological condition, or idiopathic detrusor overactivity (IDo) when there is no defined cause (1). A variety of neurological diseases that affect brain structures and spinal pathways involved in the coordination of lower urinary tract function may cause NDo, including multiple sclerosis, spinal cord injury (SCI), meningomyelocele (MMC), stroke, cerebral palsy, etc. overactive bladder syndrome (OAB) is a symptom complex including urgency, with or without urge incontinence, but usually with frequency and nocturia. This symptom combination is suggestive of detrusor overactivity which can be demonstrated by urodynamics, but it can also be due to other forms of urethrovesical dysfunction.

No matter which aetiology has caused the symptoms, the treatment of oAB has mainly relied on anticholinergic medication in an attempt to block the parasympathetic innervation of the bladder. however, although parasympatholytics have a well documented effect on oAB as well as Do, troublesome side effects are often seen, which may preclude their use at a high enough dosage or result in reduced patient compliance. Consequently, a wide variety of drugs has been or is currently under evaluation for the treatment of oAB. Also non-pharmacological modalities, including neuromodulation and surgery, have been considered and may yet prove successful, but they are not in widespread use (2-4).

For more than 25 years local injection of botulinum toxin (BT) has provided extraordinary clinical benefits in the treatment of a large variety of clinical conditions characterized by inappropriately contracting muscles. The use of BT in urology was pioneered in the eighties with injection into the urethral sphincter, and during recent years the use of BT has revolutionized the treatment of intractable Do symptoms, while pelvic pain syndrome, benign prostatic diseases and bladder pain have also been included in possible indications for BT treatment (5). In the following, a review of the applicability of BT in the treatment of NDo is presented. The review is based on a systematic search in the PubMed database until May 2011 using the medical subject headings “Botulinum Toxins, Type A”[MeSh] and “Urinary Bladder, Neurogenic”[MeSh] with the following limitations: “humans, Meta-Analysis, Randomized Controlled Trial, Review, Controlled Clinical Trial, Guideline, Journal Article, Multicenter Study, English”. The reference lists of the review and original papers were reviewed to identify missing papers.

## 2. Botulinum Toxin

Remarkably, botulism was accurately and thoroughly described by Justinus Kerner at the beginning of the nineteenth century, although it was not until late in the same century that Van Ermengem described the bacterial basis, isolated and grew the causative anaerobic bacterium and described its toxin (5-7). During the following decades the toxin was isolated and described in greater detail, in addition the requirement for toxin inactivation by heat was identified. The existence of different BT types, called A and B, were discovered at the end of World War I, and to date 7 different types, designated by the letters A to G (BT-A to BT-G) are known. Initial clinical studies on the use of BT in humans were done in 1977 on strabismus, and the FDA approved its use in adult strabismus and blepharospasm in 1989. Since then the toxin has been utilized in a surprisingly wide range of clinical conditions, including cerebral palsy, dystonia, torticollis, migraine, hyperhidrosis, oAB, etc. and has found a further extensive use in aesthetic dermatology (3, 5, 8-10).

For clinical use, therapeutic BT preparations consist of the BT component and excipients. The BT component is formed from botulinum neurotoxin (BNT) and non-toxic proteins also known as complexing proteins. BNT consists of a heavy amino acid chain with a molecular weight of 100 kD and a light amino acid chain with a molecular weight of 50 kD. These amino acid chains are interconnected by a disulfide bridge, which is essential for BT’s biological activity, thus making BT a compound which is highly vulnerable to various environmental influences. BNT and complexing proteins form BT with a molecular weight of 450 kD (11).

When BT is injected into tissues that require treatment, it binds to glycoprotein structures on the cholinergic nerve endings. here the light amino acid chain is internalized and cleaves proteins in the acetylcholine (ACh) transport chain, serving for the transportation of ACh vesicles from the intracellular space to the synaptic cleft. Which proteins are affected depends on the type of BT. After creating a blockade, the neuron starts forming new synapses to replace the blocked ones, a process called sprouting, eventually however the original synapses regenerate and the sprouts degenerate. hence, the BT interrupts the synaptic activity only temporarily and BT may therefore be classified more accurately as a temporary neuromodulator, and not as a neurotoxin (6, 11).

Depending on the target tissue, BT may block the neuromuscular transmission or the cholinergic autonomic transmission to sweat, tear and salivary glands. The onset of its action following injection occurs after a few days, and reaches a peak within approximately two to six weeks. The effect declines after several months, and usually has only a minimal effect after 6-12 months (7, 11-13). BT is transported centripetally by mechanisms of retrograde axonal transport, but this transport is so slow that BT is inactivated by the time it reaches the central nervous system. Passage to the central nervous system through the blood brain barrier is prevented due to BT’s molecular size; likewise a direct central nervous system effect beyond the alpha motor neuron has not been recorded after intramuscular injection. Despite its almost complete binding to the cholinergic nerve terminal, minute amounts of BT can be distributed via the blood circulation, however it can be detected clinically only when extremely high BT doses are used (11, 14-16). overall, BT-B has relatively stronger autonomic and weaker motor effects when compared with BT-A, the systemic spread of BT-B is also substantially higher and autonomic adverse effects occur more frequently even when low or intermediate BT-B doses are used (17, 18). In addition to the blockade of acetylcholine secretion, animal experiments have indicated that a BT induced blockade of transmitters is involved in pain sensation (11). This also seems to be the case in humans, as a rapid reduction of the patients’ sensation of urgency, which is related to Do, is reported following BT-A injection. Thus, besides a direct effect on detrusor motor innervation, BT-A probably also modulates intrinsic bladder reflexes through a multimodal effect on sensory pathways (3, 13).

BT is available in a number of therapeutic preparations in most countries (Botox®, Dysport®, Xeomin® (BT-A) and Myobloc® (BT-B)). All commercially available BT-A preparations were recently renamed with type specific, nonproprietary nomenclature, and Botox® is specifically called onabotulinum toxin A, Dysport® abobotulinum toxin A, Xeomin® incobotulinum toxin A and Myobloc® rimabotulinum toxin B (19). All BT-A preparations are powdered and need suspension in saline before use. Also, all preparations are produced biologically from a living strain of Clostridium botulinum bred under anaerobic conditions. For clinical use, the biological activity of each batch is measured by a mouse lethality test, and the specific rate is given in mouse units. however it is important to be aware, that the specific measures given by the individual manufacturers, even though given in mouse units, cannot be directly compared. Antibody formation against BT may present a significant problem in clinical settings. Antibodies directed against BNT (blocking antibodies) interfere with the biological activity of the toxin and may thus result in antibody induced therapy failure, whereas antibodies formed against non-toxic protein components in BT (non-neutralizing antibodies) do not interfere with the biological activity of BT. The risk of developing antibody induced therapy failure appears to be low and depends on patient related factors, the individual dose administered, the immunologic quality of the BT preparation and the interval between injections, etc. thus it is recommended that repeat injections should not be performed within 3 months from the previous one. There does not appear to be any link between cumulative dose, treatment time, or patient age (3, 11, 20).

The adverse effect profile of BT is mainly characterized by the inherent effects of BNT which can be local or systemic (3). All therapeutic BT-A preparations have similar adverse effect profiles. however, observations suggest an increased frequency of local adverse effects following abobotulinum toxin A when compared to onabotulinum toxin A (18). Adverse effects usually begin within a week after injection, and their severity and duration are related to the BT dose applied. Central nervous system side effects do not appear to have been reported. The adverse effect profile of therapeutic BT-B preparations is substantially different from the adverse effect profile of therapeutic BT-A preparations, and includes more pronounced autonomic side effects, whereas the frequency of adverse motor effects are comparable after both BT-A and BT-B treatment (21). The systemic spread of BT becomes clinically relevant only when the BT dose applied is inadvertently too high, however reports of episodes with prolonged, yet reversible, general muscle weakness following BT injection in spinal cord injuries have been reported (3, 22). The use of BT during pregnancy is contra-indicated. The few accidental BT applications that have occurred during pregnancies though, have not been shown to induce any foetal abnormalities (11). BT should not be used, or only with great caution in patients with pre-existing paresis, or disorders causing impaired neuromuscular transmission, such as amyotrophic lateral sclerosis, myasthenia gravis, etc. Increased paresis seen in patients with botulism receiving aminoglycoside antibiotics has led to warnings against using BT therapy and aminoglycosides simultaneously (11). Issues of long-term safety still remain, however BT therapy was introduced in the late 1980’s and large numbers of patients have since received BT over prolonged periods of time and no additional long-term adverse effects appear to have been reported (11).

only onabotulinum toxin A will be dealt with in the following review as significant differences exist between the various BT preparations. onabotulinum toxin A was the initial preparation to be used in urology and still seems to be the most widely used today.

## 3. Intravesical Injections of Onabotulinumtoxin A for the Management of Neurogenic Detrusor Overactivity

NDo may frequently lead to very distressing subjective symptoms of urge or incontinence, but serious and even potentially life threatening complications due to upper urinary tract deterioration may also be seen if detrusor pressures are sustained above 35 to 40 cm H2o (23). Consequently, patients suffering from NDo should be evaluated and treated properly, and in particular patients with a previously known increased risk for developing high bladder pressure, which might possibly be followed by renal failure – in particular SCI and MMC - should be examined at regular intervals during their lifetime.

A number of different treatment modalities; behavioural, pharmacological, surgical or interventional, have been applied in an attempt to facilitate urine storage at low bladder pressure (24). During recent years onabotulinum toxin A has been increasingly used in the treatment of NDo with the aim of improving urinary symptoms, to reduce the risk of developing upper urinary tract risk and to improve the patients’ quality of life. onabotulinum toxin A is mainly used as a second line treatment if antimuscarinic treatment has been determined to be unsuccessful in patients willing and capable of performing clean intermittent self/helper catheterization (CIC). The initial studies on onabotulinum toxin A in NDo were done by Dykstra et al. (25), who injected onabotulinum toxin A into the external rhabdosphincter in order to obtain a reversible chemical sphincterotomy, thereby providing a treatment for detrusor-sphincter dyssynergia. Ten years later Schurch et al. (19) published the initial successful results from injecting onabotulinum toxin A into the detrusor muscle in SCI patients suffering from NDo. Since then, a number of studies have confirmed the efficiency of onabotulinum toxin A in the treatment of NDo in adults and children (7, 10, 13, 26-32). The effect of onabotulinum toxin A’s effectiveness in urinary incontinence as well as on urodynamic parameters has been evaluated in a number of studies. Most studies have demonstrated a very high success rate in curing or reducing urinary incontinence, ranging from approximately 40 to 90 percent, with only a few studies giving inferior results. Maximum cystometric capacity, maximum detrusor pressure during the first detrusor contraction, compliance, reflex volume and decreased mean voiding pressures have also been improved. however post-void residual volume demonstrates a close correlation with the dose administered, in particular when small doses are applied, which is of significant importance when spontaneous voiding is highly desirable. however, CIC should always be considered in patients treated with onabotulinum toxin A, in particular when high doses are used (27, 29). The onset of the effect usually appears before 2 weeks, and reaches a maximum peak within 2-6 weeks, with a clinical effect maintained for approximately 6-8 months, and in some individuals up to more than a year. Repeat injections after intervals over 3 months are well documented, and in the majority of these patients the effect of repeated injection are similar or occasionally even better or more long lasting than the initial (3). Antimuscarinic treatment can be discontinued, or the dose reduced, in the majority of patients after onabotulinum toxin A treatment of NDo (29). however, details on injection technique, dosage, interval between injections, etc. are still under debate, and only a few placebo controlled, randomized studies exist which might elucidate these inherent questions.

## 4. Injection Technique 

Intravesical onabotulinumtoxin A injection may be performed under general, spinal or local anaesthesia, or intravenous sedation, this mainly depends on patient and local preferences, using a rigid or flexible cystoscope and an injection needle fitted to the applied cystoscope. The injection should be given into the detrusor muscle and care should be taken not to penetrate the thin bladder wall and inject into the perivesical tissues (3, 7, 12, 13, 29, 30, 33). In our institution this is ensured by administering a suburothelial injection, thereby creating a submucosal bleb allowing for subsequent diffusion into the detrusor muscle. All patients are given perioperative antibiotic prophylaxis.

The first report of an intravesical toxin injection described 1 ml of onabotulinum toxin A in a 10 IU/ml suspension injected directly into the detrusor muscle at 30 injecting sites, excluding the trigone (28). Since then most studies have applied similar injecting techniques, even though the number of injection sites ranged from 10 to 50, and the dilution range from 10 IU/ml to 100 IU/ ml (3, 7, 10, 13, 29, 31). The decision to avoid the trigone was multifactorial and included a desire to avoid inducing reflux in the upper urinary tract, along with concerns that injecting may affect the dense trigonal innervation from the sensory, adrenergic, and non-cholinergic pathways, and subsequent investigations have predominantly avoided trigone injections (3).

Recent research however, has suggested that sensory neuron dysfunction may actually contribute to the pathophysiology of sensory urgency and Do (3). Additionally, increasing evidence suggests that BT inhibits both sensory neuron action and the release of sensory neuropeptides from adjacent cell types (i.e. urothelium) that may contribute to sensory signaling, and this data might suggest a possible benefit for including the trigone. Studies have also reported successful outcomes following BT-A injection into the trigone (3, 13). however, no direct comparisons were made with patients receiving trigone-sparing injections. luiciano et al. (34) compared inclusion vs. exclusion of the trigone in the injection protocol, but found no difference between these techniques although only 2 of the 30 injections were actually administered into the trigone. on the other hand, recent results from a randomized, prospective study, which examined the results of injecting 300 IU onabotulinum toxin A solely into the bladder dome and excluding the trigone vs. an injection of 200 IU onabotulinum toxin A into the dome, along with a further 100 IU into the trigone showed significant differences in complete dryness in favour of the trigone injection (33).

## 5. Injection Dose

The majority of published studies have utilized a 300 IU dose in adults, but some studies report using from 100 to 400 IU onabotulinum toxin A doses (3, 7, 12, 29). In children, the most common dosage of onabotulinum toxin A is 5-12 IU/kg, with a maximum dose of 300 IU (10, 13, 31, 32). No dose-response studies were published until Schurch et al. (26) reported their results from a direct comparison of 59 patients with NDo randomized to receive injections of 200 or 300 IU of onabotulinum toxin A or a placebo. Significant subjective and objective improvement was seen in the two active arms, including improved continence, bladder capacity, and maximum detrusor pressure, but not in the placebo arm and no difference in primary outcomes were demonstrated when comparing the active arms. however, this outcome may have been affected by the rather small study sample size. Kuo et al. (27) reported a randomized comparison of an injection of 100, 150, and 200 IU onabotulinumtoxin A in the treatment of 75 patients with Do from refractory to anticholinergics. Clinical and urodynamic outcomes were similar between the 150 and 200 IU groups, with those patients receiving 100 IU experiencing less favorable therapeutic results, along with lower post-void residual volume. Based on this data, the authors conclude that a 150 IU dose provides a more satisfactory outcome with a decrease in adverse effects compared to the 200 IU dose. Unfortunately 300 IU, the most utilized dose, was not included in the study comparison. In a recent multicenter, double-blind, randomized, placebo controlled parallel study 275 patients with urinary incontinence due to NDo from SCI or MS were randomized to receive 30 intradetrusor injections with 300 IU onabotulinum toxin A, 200 IU onabotulinum toxin A or a placebo, avoiding the trigone (35). Results showed that the frequency of urinary incontinence episodes was significantly reduced in both the 300 IU and 200 IU onabotulinum toxin A groups when compared to the placebo, whereas no difference was demonstrated between the active groups. Almost 40 percent of the onabotulinum toxin A treated patients were reported to be continent. Duration of the effect was 295 days. median Post-void residual volume, however, increased after the onabotulinum toxin A injection and it was significantly higher in the 300 IU onabotulinum toxin A group.

## 6. Adverse Effects 

Most patients tolerate onabotulinum toxin A treatment of NDo well. The most frequently reported local problems are; injection site pain, urinary tract infection and mild haematuria (3, 7, 13, 19, 29, 30). Post-void residual volume may also frequently present a challenge, which can easily be solved by CIC (13, 29). The beneficial effects of the toxin remain after repeated injections, and the risk of developing bladder fibrosis following frequent treatment with BT-A has not been confirmed (5, 13, 19, 36-38).

Generalized muscle weakness has been reported, but only casuistically, and even though highly alarming when it occurs, this reverses simultaneously with the effects of the onabotulinum toxin A, and furthermore it does not seem to present a frequent problem (3, 22). other systemic side effects include flu-like symptoms, dry mouth and general malaise. Finally, it is evident that manipulations of the lower urinary tract always includes an increased risk of autonomic dysreflexia in SCI-patients with lesions above Th6 (13).

## 7. Conclusions

For more than 25 years onabotulinum toxin A has found widespread use in urology. Toxin treatment is easily performed and has proved to have a favourable side effect profile. In patients suffering from NDo, onabotulinum toxin A has mainly been used as a second line treatment after antimuscarinic treatment has failed, and in these patients onabotulinum toxin A has proved highly effective in improving urinary symptoms, reducing the risk of developing upper urinary tract complications and improving quality of life. Recent data from randomized controlled studies indicates that the use of 200 UI onabotulinum toxin A results in an optimal reduction in Do. Intervals between repeated treatments should be longer than 3 months. Treatment may induce a significant risk for increased urinary residual volume, however this is easily treated by CIC. The optimal injection protocol, including the number of injection sites, and whether or not to include the trigone, has not yet been determined.
